# The long-term hospitalization experience following military service in the 1991 Gulf War among veterans remaining on active duty, 1994–2004

**DOI:** 10.1186/1471-2458-8-60

**Published:** 2008-02-13

**Authors:** Tomoko I Hooper, Samar F DeBakey, Barbara E Nagaraj, Kimberly S Bellis, Besa Smith, Tyler C Smith, Gary D Gackstetter

**Affiliations:** 1 Department of Preventive Medicine and Biometrics, Uniformed Service University of the Health Sciences, Bethesda, Maryland, USA; 2 Health Research and Analysis, Rockville, Maryland, USA; 3 Department of Defense Center for Deployment Health Research, Naval Health Research Center, San Diego, California, USA; 4 Analytic Services, Inc., Arlington, Virginia, USA; 5 Henry M. Jackson Foundation for the Advancement of Military Medicine, Inc., Rockville, Maryland, USA; 6 Altarum Institute, Alexandria, VA, USA

## Abstract

**Background:**

Despite more than a decade of extensive, international efforts to characterize and understand the increased symptom and illness-reporting among veterans of the 1991 Gulf War, concern over possible long-term health effects related to this deployment continue. The purpose of this study was to describe the long-term hospitalization experience of the subset of U.S. Gulf War veterans still on active duty between 1994 and 2004.

**Methods:**

Gulf War veterans on active duty rosters as of October 1, 1994, were identified (n = 211 642) and compared with veterans who had separated from military service and then assessed for attrition at three-year intervals during a 10-year follow-up period, examining demographic and military service characteristics, Gulf War exposure variables, and hospitalization data. Cox proportional hazard modeling was used to evaluate independent predictors of all-cause hospitalization among those still on active duty and to estimate cumulative probability of hospitalization, 1994–2004, by service branch.

**Results:**

Members of our 1994 active duty cohort were more likely to be officers, somewhat older, and married compared with those who had separated from the military after serving in the 1991 Gulf War. Selected war-related exposures or experiences did not appear to influence separation with the exception of in-theater presence during the brief ground combat phase. Overall the top three diagnostic categories for hospitalizations were musculo-skeletal, injury and poisoning, and digestive disorders. Diseases of the circulatory system and symptoms, signs, and ill-defined conditions increased proportionately over time. In-theater hospitalization was the only significant independent predictor of long-term hospitalization risk among selected war-related exposures or experiences examined. The cumulative probability of hospitalization was highest for Army and lowest for Marines.

**Conclusion:**

Our results were generally consistent with a previous hospitalization study of US Gulf War veterans for the period August 1991 to July 1999. Although lack of a comparison group for our study limits interpretation of overall findings, intra-cohort analyses showed no significant associations between long-term hospitalization and war-related exposures or experiences, with the exception of in-theater hospitalization, within our active duty subset of 1991 Gulf War veterans.

## Background

Concern for the health of individuals reporting symptoms or illnesses following service in the 1991 Gulf War led to wide-ranging, international investigations for over more than a decade that attempted to characterize a unique syndrome and/or identify possible deployment-related causes [1-23]. Some specific exposures investigated include smoke from oil well fires [24-28], nerve agents released during munitions demolition at Khamisiyah, Iraq [28-38], immunizations [12,39-48], pesticides [41,44,45,47,49-54], chemical warfare nerve agent prophylaxis [45,47,52,53,55-60], and other exposures (single or multiple), including psychological stressors [41,44,50,52,53,58,61-73]. While several researchers have reported associations between some Gulf War exposures and specific health effects [34,36,41,56], epidemiologic studies to date have not provided strong and consistent evidence for a causal relationship between putative exposures and the full array of symptoms and illnesses associated with deployment to the 1991 Gulf War [19,66,68,74-80].

Previous studies have examined the early postwar hospitalization experience of U.S. Gulf War veterans compared with non-deployed era veterans and found no unexplained differences for most diagnostic categories [81,82]. Excess hospitalizations among Gulf War veterans for some diagnoses were most often viewed as a consequence of deferred treatment, postwar adjustment to the stresses of war, or participation in a Gulf War health registry program with admission to hospital for advanced diagnostic procedures [81-85]. Investigations of the association between specific environmental exposures and hospitalization among deployed Gulf War veterans have not demonstrated increased overall risk among those exposed [26,28,29,32,33]. Lastly, longitudinal studies of mortality among U.S. and U.K. Gulf War veterans have not shown significant differences in overall risk of death due to disease among those deployed to the Gulf compared with non-deployed comparison groups [30,54,86-88]. Excess deaths due to unintentional injury (primarily motor vehicle crashes) among Gulf War veterans were observed for up to seven years post-war, but subsequently became comparable to the risk among non-deployed [30,54]. However, a recent report suggested increased cause-specific risk of death from brain cancer among Gulf War veterans potentially exposed to munitions demolition at Khamisiyah [34].

Thus, concern over possible long-term, adverse health outcomes from war-related exposures has persisted more than 15 years since the end of the 1991 Gulf War. Our study focuses on a subset of Gulf War veterans who were on active duty as of October 1, 1994, and describes their long-term hospitalization experience in three-year intervals over the decade from 1994 to 2004. The purpose was to characterize this active duty subset over time and examine hospitalization as an objective measure of long-term morbidity. We evaluated the effect of selected war-related exposures or experiences on hospitalization by service branch, while adjusting for demographic and military service characteristics.

## Methods

All regular active duty US military personnel deployed to the 1991 Gulf War (n = 584 341) were identified by the Defense Manpower Data Center (DMDC), Seaside, California, USA. We examined attrition over time to select a point at which approximately 50% of this population was still on active duty, to allow for the usual troop reductions after a large scale combat operation and to focus on men and women more likely to represent those continuing a military career. Among those deployed to the Gulf War, 265 690 were still listed on active duty rosters as of October 1, 1994. Of this group, 1281 had no DMDC record available for the Gulf War period, leaving 264 409 in our study population.

Demographic and military service characteristics, selected Gulf War exposure variables, and Department of Defense (DoD) hospitalization data were obtained from databases maintained at DMDC, the Naval Health Research Center (NHRC), San Diego, California, USA, and the US Army Center for Health Promotion and Preventive Medicine (CHPPM), Aberdeen Proving Ground, Maryland, USA. DMDC provided demographic and military service data, and the following variables were used to characterize our study population at the time of the Gulf War and at three-year intervals during the follow-up period: age (16–22, 23–26, 27–31, 32–61; quartiles based on distribution as of October 1, 1994), gender, marital status (single, married, other), race (black, white, other), service (Army, Navy, Air Force, Marine Corps), and pay grade (officer, enlisted). In addition, military occupation was categorized into 10 groups based on the DoD 2001 Occupational Conversion Index [89], and for multivariable modeling, dichotomized into combat specialists versus other. We used wartime demographic and military profiles as the basis for observing changes in our active duty cohort and as covariates in time-to-event modeling.

Available environmental exposure data from NHRC and CHPPM included information on potential exposure to munitions demolition at Khamisiyah (March 10–12, 1991) and smoke from oil well fires. The basis for establishing exposure status with respect to Khamisiyah and oil well fires has been previously described [26,33,90]. For purposes of multivariable modeling, we used a three-level Khamisiyah exposure variable (presumed not at risk; at risk of exposure but not exposed; and exposed). Other war-related exposures or experiences, including anthrax and/or botulinum immunization, in-theater hospitalization, and presence in theater during ground combat operations (February 24–28, 1991) were all treated as dichotomous variables.

We assessed the distribution of wartime demographic and military characteristics and deployment-related exposure variables at the start of four time intervals (1995–1997, 1998–2000, 2001–2003, and 2004) based on the federal fiscal year (FY), starting October 1 and ending September 30. For example, FY 1995 corresponds to October 1, 1994, through September 30, 1995. We then evaluated hospitalizations during the entire 10-year period of observation (October 1, 1994, to September 30, 2004), as well as over each time interval, to identify the most frequently diagnosed conditions and any observable trends. To reduce possible effects of other deployments, we excluded any service member who deployed between the end of the Gulf War and the beginning of our period of observation (n = 49 502). After further excluding individuals with missing covariate data (n = 3265), our final analytic data set for multivariable modeling (time-to-event) included 211 642 individuals.

Hospitalization data between October 1, 1994, and September 30, 2004, consisted of inpatient encounter records from military treatment facilities, as well as civilian facilities when costs were reimbursed by the DoD. Up to eight discharge diagnoses are recorded for each hospitalization based on *International Classification of Diseases, 9th Revision, Clinical Modification *(ICD-9-CM) codes. We included 14 major ICD-9-CM diagnostic categories related to illness or injury in our analysis, excluding congenital anomalies and pregnancy-related categories. In order to capture the full array of medical conditions for which individuals might be hospitalized, we considered both primary and secondary diagnoses. Individuals could be counted in multiple diagnostic categories, but just once per category. Additionally, in-theater hospitalization data for individuals in our study population were also available for analysis.

We used Cox proportional hazards modeling to evaluate independent predictors of all-cause hospitalization, while adjusting for covariates and accounting for varying lengths of follow-up. Initial data exploration included bivariate analyses, Kaplan-Meier survival analysis, and the log-rank test for equality across strata to assess proportionality over time for each covariate, followed by regression diagnostics to assess collinearity. Finally, potential confounding and effect modification were assessed through exploratory time-to-event modeling. All covariates were considered eligible for inclusion in the final Cox regression model if they did not exhibit collinearity and met the assumption of proportional hazard over time. Variables related to immunization, oil well fire smoke exposure, potential nerve agent exposure, and presence in theater during ground combat were all retained as covariates, despite lack of significance, to address ongoing general public and veteran concerns and to quantify the influence of these exposures. We modeled the number of months from October 1, 1994, until first hospitalization for any cause. Observations were censored at separation from military service, death, subsequent deployment (Bosnia, Kosova, or Southwest Asia), or end of study period, whichever occurred first. Analyses were performed using SAS^® ^software, version 9.1 (SAS Institute, Cary, North Carolina, USA).

The protocol for this study was reviewed and approved by the Institutional Review Board at the Uniformed Services University of the Health Sciences and the Naval Health Research Center and was conducted in compliance with all applicable federal regulations governing the protection of human subjects in research.

## Results

Table 1 compares Gulf War veterans in our active duty study population at the beginning of the observation period with those who had separated from military service prior to October 1, 1994. Our active duty subset tended to be slightly older, married, and officers. Gender and race were comparable between the two groups. Among the service branches, there were some notable differences, with Navy and particularly Air Force personnel tending to remain on active duty and Army and Marine Corps personnel more likely to have separated. Comparing occupational categories, those in electrical/mechanical repair were more likely to remain on active duty in contrast to those in electronics repair and combat specialists, who were more likely to have separated from military service. For war-related exposures, there were no substantial differences between those who stayed on active duty and those who separated, except for presence in the theater of operations during the five days of ground combat. Overall, about 85% of deployed service members were in theater during ground combat (data not shown). By October 1, 1994, about 78% of those still on active duty had been in theater during this combat period compared with 91% of those who had separated from military service.

**Table 1:** Distribution of wartime demographic and military characteristics of personnel who served in the 1991 Gulf War and remained on active duty, FY 1995–2004, compared with those who separated prior to FY 1995

War-time characteristic	Separated	Remained on active duty			
	
	<FY 1995	FY 1995	FY 1998	FY 2001	FY 2004
	
	%	%	%	%	%
Total service members with wartime demographic profile available	*n *= 321 806	*n *= 264 409	*n *= 179 216	*n *= 128 544	*n *= 101 591
**Gender**					
Male	93.8	93.8	93.9	94.0	93.9
Female	6.1	6.2	6.1	6.0	6.1
Unknown	0.1	0.0	0.0	0.0	0.0
**Age (years)**					
16–22	35.2	26.1	23.5	24.1	28.3
23–26	29.8	24.2	26.7	30.5	34.3
27–31	18.0	25.4	30.1	30.0	25.3
32–61	16.9	24.3	19.7	15.5	12.1
Unknown	0.2	0.0	0.0	0.0	0.0
**Marital status**					
Single, never married	56.0	35.6	34.5	36.1	40.2
Married	42.8	61.7	62.8	61.3	57.4
Other	0.9	2.8	2.7	2.5	2.3
Unknown	0.3	0.0	0.0	0.0	0.0
**Race**					
Black	21.9	24.9	25.4	25.1	25.4
White	68.6	67.7	66.9	67.1	66.6
Other	9.2	7.3	7.6	7.8	7.9
Unknown	0.2	0.1	0.1	0.1	0.1
**Service**					
Army	49.3	43.5	42.2	41.8	43.0
Navy*	23.8	28.2	28.0	27.9	27.2
Air Force	8.7	16.2	17.6	17.9	17.4
Marine Corps	18.3	12.2	12.2	12.4	12.4
**Pay grade**					
Commissioned officer	6.0	13.4	14.7	16.1	16.7
Warrant officer	0.8	2.0	1.9	1.8	1.6
Enlisted	93.2	84.7	83.3	82.2	81.7
Unknown	0.0				
**Primary military occupation grouping**					
Combat specialties	28.8	23.7	24.5	25.2	25.9
Communications, intelligence	10.6	9.9	9.9	9.9	9.8
Craftsworkers	3.6	3.4	3.3	3.2	3.2
Electronics repair	19.6	9.5	9.3	9.0	8.7
Electrical/mechanical repair	7.6	20.4	20.0	19.9	19.6
Functional support	10.4	12.3	12.2	12.1	11.9
Health care specialists	4.5	6.5	6.7	6.7	6.8
Non-occupational	1.3	2.7	2.6	2.6	2.8
Other technical specialists	2.0	2.3	2.4	2.3	2.1
Service support	10.6	9.2	9.1	9.1	9.2
Unknown	1.0	0.0	0.0	0.0	0.0
**War-related exposures**					
Anthrax immunization	0.4	0.4	0.4	0.4	0.4
Botulinum immunization	(<0.1)	0.4	0.4	0.4	0.4
Exposed to Khamisiyah March 10–12, 1991	13.7	12.5	12.2	12.1	12.4
Exposed to oil well fire 1991	65.1	61.0	61.0	61.0	61.4
In-theater hospitalization	2.9	2.1	2.1	2.0	2.0
In theater during combat period	90.9	78.1	78.3	78.5	78.8
**Returned to the Gulf between end of war and FY 1995**		18.7	18.9	17.5	18.6
**Subsequent deployment (Bosnia, Kosovo, SW Asia)**		15.0	20.0	18.8	21.2

FY: Fiscal year (October 1 through September 30)

* Includes Coast Guard.

Table 1 also shows the distribution of demographic and military service characteristics at three-year time intervals for those remaining on active duty. With the exception of age, the proportions remained relatively consistent over time, including service branch. As expected, the oldest age category was less represented in the population remaining on active duty over time.

Table 2 shows the distribution of ICD-9-CM diagnostic categories (including primary and secondary diagnoses) for inpatient encounters during the 10 years of follow-up. The most frequent categories were diseases of the musculoskeletal system (32.7%); digestive system (23.4%); injury and poisoning (21.0%); symptoms, signs, and ill-defined conditions (18.7%); circulatory conditions (14.7%); mental disorders (13.8%); and respiratory conditions (12.2%). We also examined the top five primary diagnoses within each of the 14 diagnostic categories over the entire follow-up period (Table 3). The following briefly describes the proportion of individuals with the most common primary diagnoses within the categories mentioned above. For musculoskeletal system diseases, 24.5% had a diagnosis of internal derangement of the knee and 18.3% had intervertebral disc disorders; within digestive diseases, 17.3% had inguinal hernia and 11.8% acute appendicitis. Within the injury and poisoning category, 9.2% had complications of procedures, 7.7% ankle fractures, and 7.0% sprains and strains of the knee and leg. For ill-defined conditions, 46.6% had respiratory system and other chest symptoms, and 22.3% had general symptoms. Within diseases of the circulatory system, 15.5% had cardiac dysrhythmias, 14.8% other chronic ischemic heart disease, and 13.5% hemorrhoids, while for respiratory system diseases, 24.3% had deviated nasal septum, 12.1% unspecified pneumonia, and 11.7% chronic disease of tonsils and adenoids. Finally, in the category of mental disorders, 33.1% had alcohol dependence syndrome and 29.6% adjustment reaction.

**Table 2:** Distribution of both primary and secondary diagnoses within 14 ICD-9-CM diagnostic categories for hospitalized individuals who served in the 1991 Gulf War and remained on active duty, FY 1995–2004

	All years	FY 1995–1997	FY 1998–2000	FY 2001–2003	FY 2004
		
	*n *= 43 346	*n *= 30 629	*n *= 10 588	*n *= 7572	*n *= 1937
Diagnostic categories*	**%**^†^	**%**	**%**	**%**	**%**

Infectious and Parasitic Diseases (001–139)	7.0	5.5	6.4	6.3	6.7
Neoplasms (140–239)	7.5	6.2	6.2	7.9	8.8
Endocrine, Nutritional and Metabolic Diseases, and Immunity Disorders (240–279)	10.4	5.8	9.4	14.7	16.9
Diseases of the Blood and Blood-Forming Organs (280–289)	5.4	3.3	5.9	6.2	5.3
Mental Disorders (290–319)	13.8	10.4	13.3	14.0	14.8
Diseases of the Nervous System and Sense Organs (320–389)	7.5	6.8	5.3	5.4	5.8
Diseases of the Circulatory System (390–459)	14.7	9.4	14.3	20.8	25.1
Diseases of the Respiratory System (460–519)	12.2	11.0	9.3	9.4	9.7
Diseases of the Digestive System (520–579)	23.4	20.1	19.6	22.4	23.4
Diseases of the Genitourinary System (580–629)	11.1	9.5	9.6	10.0	11.5
Diseases of the Skin and Subcutaneous Tissue (680–709)	5.4	4.8	3.8	4.5	4.1
Diseases of the Musculoskeletal System and Connective Tissue (710–739)	32.7	32.1	25.6	26.0	26.1
Symptoms, Signs, and Ill-Defined Conditions (780–799)	18.7	13.0	17.1	23.2	24.2
Injury and Poisoning (800–999)	21.0	16.6	20.8	20.9	19.6

ICD-9-CM: *International Classification of Diseases, 9th Revision, Clinical Modification*.

FY: Fiscal year (October 1 through September 30).

* Each individual could only be counted once (primary or secondary diagnosis) within a particular diagnostic category, but could be counted in multiple categories.

^† ^Percentages do not add to 100% because hospitalization for each individual could be counted in more than one category.

**Table 3:** Top five primary diagnoses within 14 major ICD-9-CM diagnostic categories for hospitalized individuals who served in the 1991 Gulf War and remained on active duty, FY 1995–2004

**DIAGNOSTIC CATEGORY**	**n**	**%**	**DIAGNOSTIC CATEGORY**	**n**	**%**
**Infectious and Parasitic Diseases **(n = 1115)			**Diseases of the Respiratory System **(n = 2872)		
Intestinal infections due to other organisms	140	12.6	Deviated nasal septum	698	24.3
Viral infection in conditions classified elsewhere and of unspecified site	140	12.6	Pneumonia, organism unspecified	347	12.1
Meningitis due to enterovirus	138	12.4	Chronic disease of tonsils & adenoids	337	11.7
Viral hepatitis	103	9.2	Other diseases of upper respiratory tract	301	10.5
Sarcoidosis	79	7.1	Chronic sinusitis	255	8.9
**Neoplasms **(n = 2106)			**Diseases of the Digestive System **(n = 7446)		
Uterine leiomyoma	483	22.9	Inguinal hernia	1290	17.3
Lipoma	217	10.3	Acute appendicitis	877	11.8
Benign neoplasm of bone and articular cartilage	112	5.3	Diseases of esophagus	835	11.2
Malignant neoplasm of prostate	94	4.5	Dentofacial anomalies, including malocclusion	776	10.4
Benign neoplasm of other parts of digestive system	91	4.3	Cholelithiasis	453	6.1
**Endocrine, Nutritional and Metabolic Diseases, and Immunity Disorders **(n = 707)			**Diseases of the Genitourinary System **(n = 2964)		
Diabetes mellitus	219	31.0	Calculus of kidney & ureter	577	19.5
Disorders of fluid, electrolyte, & acid-base balance	114	16.1	Other disorders of breast	259	8.7
Disorders of lipoid metabolism	69	9.8	Other disorders of male genital organs	231	7.8
Nontoxic nodular goiter	56	7.9	Pain & other symptoms assoc with female genital organs	225	7.6
Obesity & other hyperalimentation	54	7.6	Disorders of menstruation & other abnormal bleeding from female genital tract	142	4.8
**Diseases of the Blood and Blood-Forming Organs **(n = 204)			**Diseases of the Skin and Subcutaneous Tissue **(n = 1192)		
Other diseases of blood & blood-forming organs	54	26.5	Other cellulitis & abscess	559	46.9
Diseases of white blood cells	53	26.0	Pilonidal cyst	115	9.6
Purpura & other hemorrhagic conditions	33	16.2	Other disorders of skin & subcutaneous tissue	105	8.8
Iron deficiency anemias	24	11.8	Other hypertrophic & atrophic conditions of skin	97	8.1
Other & unspecified anemias	23	11.3	Diseases of sebaceous glands	85	7.1
**Mental Disorders **(n = 3358)			**Diseases of the Musculoskeletal System and Connective Tissue **(n = 11 908)		
Alcohol dependence syndrome	1112	33.1	Internal derangement of knee	2920	24.5
Adjustment reaction	995	29.6	Intervertebral disc disorders	2179	18.3
Affective psychoses	689	20.5	Other derangement of joint	1302	10.9
Neurotic disorders	195	5.8	Other disorders of synovium, tendon, & bursa	1042	8.8
Depressive disorder, not elsewhere classified	170	5.1	Peripheral enthesopathies & allied syndromes	969	8.1
**Diseases of the Nervous System and Sense Organs **(n = 1731)			**Symptoms, Signs, and Ill-Defined Conditions **(n = 4031)		
Mononeuritis of upper limb & mononeuritis multiplex	301	17.4	Symptoms involving respiratory system & other chest symptoms	1880	46.6
Migraine	180	10.4	General symptoms	900	22.3
Mononeuritis of lower limb	163	9.4	Other symptoms involving abdomen & pelvis	481	11.9
Disorders of conjunctiva	72	4.2	Other ill-defined & unknown causes of morbidity & mortality	241	6.0
Other disorders of eyelids	71	4.1	Symptoms involving head & neck	178	4.4
**Diseases of the Circulatory System **(n = 2652)			**Injury and Poisoning **(n = 6416)		
Cardiac dysrhythmias	412	15.5	Other complications of procedures, not elsewhere classified	589	9.2
Other forms of chronic ischemic heart disease	393	14.8	Fracture of ankle	495	7.7
Hemorrhoids	359	13.5	Sprains & strains of knee & leg	452	7.0
Acute myocardial infarction	320	12.1	Complications peculiar to certain specified procedures	431	6.7
Varicose veins of lower extremities	144	5.4	Sprains & strains of ankle & foot	279	4.3

**Figure 1:**
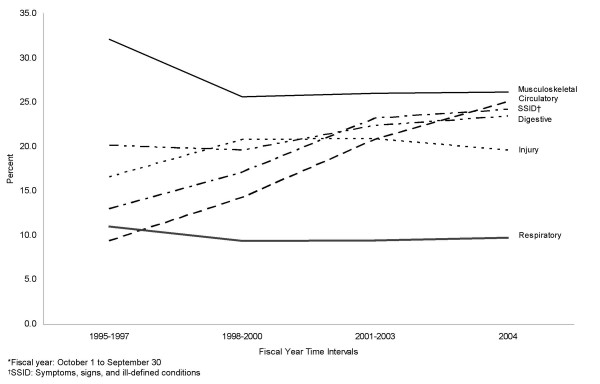
Changes in relative proportions of selected diagnostic categories for hospitalized individuals who served in the 1991 Gulf War and remained on active duty, fiscal years* 1995 to 2004.

Examining hospitalizations at three-year intervals, the leading diagnostic categories remained consistent over time, although some changes occurred in rank order, and diseases of the circulatory system gained prominence, increasing from 9.4% in the FY 1995–1997 time interval to 25.1% in FY 2004 (Table 2 and Figure 1). Similar proportional increases occurred over time for endocrine, metabolic, and immunity disorders; mental disorders; and symptoms, signs, and ill-defined conditions.

There were 4800 in-theater hospitalization records for our study cohort. Overall, the most frequently encountered diagnostic categories (data not shown) were injury and poisoning (28.3%); diseases of the digestive system (19.4%); symptoms, signs, and ill-defined conditions (12.4%); and diseases of the musculoskeletal system and connective tissue (12.3%). Except for differences in rank order, these were consistent with the top four diagnostic categories for hospitalizations during the entire follow-up period of our study.

**Figure 2:**
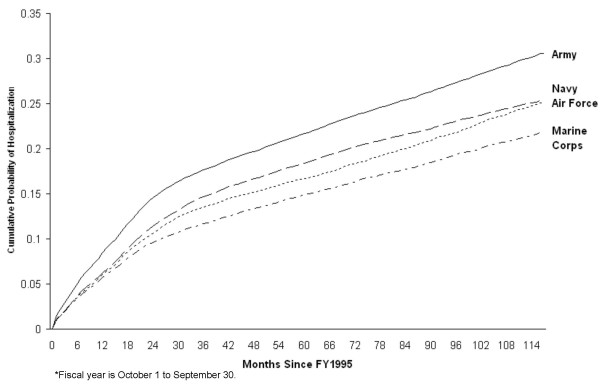
Cumulative probability of first hospitalization for any cause by service branch among military personnel who served in the 1991 Gulf War and remained on active duty, fiscal years* 1995 to 2004.

Time-to-event modeling was stratified by service branch because the assumption of proportionality over time was not met. The cumulative probability of first hospitalization is presented in Figure 2. Long-term hospitalization risk was highest for Army (31% over 9.8 years) and lowest for Marine Corps (22% over 9.7 years). Adjusted risk ratios for selected covariates are provided in Table 4. Female gender and being married were independent predictors of hospitalization for all services, and hospitalization risk increased with age. There was some variability among the service branches with respect to race and hospitalization risk. For Army, those categorized as white race had a slightly increased risk compared with black race.

**Table 4:** Adjusted risk ratios by service branch using Cox proportional hazards modeling for postwar all-cause hospitalization among military personnel who served in the 1991 Gulf War and remained on active duty, FY 1995–2004

	Army		Navy		Air Force		Marine Corps	
	
Covariate	RR*	95% CI ^†^	RR	95% CI	RR	95% CI	RR	95% CI
**Gender**								
Female	1.70	(1.63, 1.77)	1.94	(1.78, 2.11)	1.83	(1.67, 2.01)	2.23	(1.89, 2.64)
Male	Ref.		Ref.		Ref.		Ref.	
**Age (years)**								
23–26	1.02	(0.98, 1.07)	1.02	(0.95, 1.10)	1.01	(0.91, 1.11)	1.24	(1.13, 1.37)
27–31	1.13	(1.08, 1.18)	1.13	(1.05, 1.21)	1.15	(1.04, 1.26)	1.49	(1.35, 1.65)
32–61	1.51	(1.44, 1.58)	1.51	(1.41, 1.63)	1.35	(1.21, 1.50)	2.02	(1.82, 2.24)
16–22	Ref.		Ref.		Ref.		Ref.	
**Marital status**								
Married	1.06	(1.02, 1.10)	1.15	(1.09, 1.21)	1.10	(1.02, 1.18)	1.14	(1.06, 1.23)
Other	Ref.		Ref.		Ref.		Ref.	
**Race**								
Black	0.92	(0.89, 0.95)	0.98	(0.92, 1.03)	1.00	(0.92, 1.09)	0.98	(0.91, 1.06)
Other^‡^	0.91	(0.87, 0.97)	0.71	(0.65, 0.78)	0.83	(0.70, 0.97)	0.91	(0.80, 1.04)
White	Ref.		Ref.		Ref.		Ref.	
**Pay grade**								
Enlisted	0.63	(0.60, 0.66)	0.70	(0.64, 0.75)	0.86	(0.78, 0.94)	0.78	(0.71, 0.86)
Officer	Ref.		Ref.		Ref.		Ref.	
**War-related exposures**								
Anthrax/botulinum immunization^§^								
Yes	1.04	(0.88, 1.22)			0.91	(0.65, 1.28)		
No	Ref.		Ref.		Ref.		Ref.	
Exposed to Khamisiyah March 10–12, 1991^#^								
Yes (at risk and exposed)	1.03	(0.94, 1.12)			1.01	(0.89, 1.14)		
No (at risk and not exposed)	0.99	(0.91, 1.08)			1.06	(0.83, 1.35)		
Never at risk	Ref.		Ref.		Ref.		Ref.	
Exposed to oil well fire 1991								
Yes	1.03	(0.99, 1.07)	0.97	(0.92, 1.02)	0.96	(0.90, 1.03)	1.04	(0.96, 1.13)
No	Ref.		Ref.		Ref.		Ref.	
In-theater hospitalization								
Yes	1.60	(1.49, 1.71)	1.71	(1.41, 2.07)	1.50	(1.31, 1.73)	1.78	(1.48, 2.14)
No	Ref.		Ref.		Ref.		Ref.	
In theater during combat period								
Yes	0.94	(0.86, 1.03)	1.07	(1.02, 1.13)	0.96	(0.84, 1.09)	0.99	(0.90, 1.09)
No	Ref.		Ref.		Ref.		Ref.	
Occupation								
Combat specialists	1.01	(0.97, 1.04)	1.05	(0.99, 1.12)	0.82	(0.74, 0.92)	0.92	(0.86, 0.99)
Other	Ref.		Ref.		Ref.		Ref.	

FY: Fiscal year (October 1 through September 30).

* RR: risk ratio; adjusted for all other covariates in the model.

^† ^CI: Confidence interval.

^‡ ^Other: heterogeneous group, including Asian American, Hispanic, and Native American, among others.

^§ ^Immunizations data not complete.

^# ^Only Army and Air Force data available for Khamisiyah munitions demolition exposure.

Other independent predictors of hospitalization consistent across services included enlisted rank and history of in-theater hospitalization. Enlisted rank was associated with a reduced risk of hospitalization, whereas a history of in-theater hospitalization increased subsequent long-term hospitalization risk. Presence in theater during ground combat was a predictor of hospitalization for Navy only, and combat specialists in the Air Force and Marine Corps (but not Army and Navy) were at reduced risk of hospitalization compared with other occupational groups.

## Discussion

Understanding the complex, interrelated health issues associated with the 1991 Gulf War has proved challenging and is not completely resolved. Our study focused on describing the hospitalization experience of those who were still on active duty at least three years following the 1991 Gulf War because of interest in retention and resiliency issues. We characterized those with career longevity and examined hospitalization as an objective measure of morbidity in this subgroup for up to 13 years following service in the Gulf War.

Demographic and military characteristics appeared to have influenced separation from military service more than specific war-related exposures or experiences, with the exception of presence in theater during the brief ground combat phase. How these characteristics influence separation may well represent a predictable exodus of young, single, infantry personnel leaving the military following any large-scale combat operation, since significant attrition is not surprising as an aftermath of a large military build-up. In addition, evolving career goals, especially among young recruits completing an initial tour of military duty, may also play an important role in losses from the military. On the other hand, service members who had negative experiences or poor health related to deployment may have been more likely to separate from military service and been lost to follow-up.

The total number of individuals hospitalized at least once over the 10-year period of observation was 43 346 (16.4% of the 264 409 active duty Gulf War veterans assembled for follow-up at the start of FY 1995). This proportion is somewhat less than the 19.4% of active duty Gulf War veterans previously reported hospitalized at least once between August 1, 1991, and July 31, 1999 [21,26]. Fitness for duty standards clearly result in some premature separations from military service due to serious medical conditions, both physical and mental. However, it is possible that the demographics of our subpopulation with career longevity may be indicative of a healthier occupational cohort due to social and behavioral factors, notwithstanding the expected increase in chronic medical conditions related to advancing age over time.

Hospital discharge diagnoses (primary or secondary) among individuals hospitalized in the current study most frequently fell within categories related to musculoskeletal, digestive, injury, ill-defined, and circulatory conditions (Table 2). This was not surprising for a cohort with nearly 25% age 32 years or older at baseline (and nearly 50% 27 years or older) and aging over the 10-year period of observation. If we consider as a point of reference, the top five diagnostic categories for hospitalizations among all U.S. Armed Forces during comparable time periods, the only notable difference was in the category of mental disorders, which was consistently under-represented in our study population. It is noteworthy that for all of DoD, mental disorders ranked among the top two major diagnostic categories (excluding pregnancy-related conditions) between 2000 and 2004 [91], while for our Gulf War-deployed, active duty subset, mental disorders did not rank among the top five. This probably reflects some attrition related to serious psychiatric disorders, as well as proportional increases in those chronic conditions associated with aging. For example, circulatory conditions steadily increased over the study period, as did diabetes, which was the primary underlying basis for the increase over time in the category of endocrine, nutritional, and metabolic diseases.

Musculoskeletal conditions were the most frequent hospital diagnoses over the 10-year period of observation, which was similar to the distribution for DoD-wide hospitalizations (excluding pregnancy-related conditions) until more recent years, when DoD numbers for this diagnostic category showed a relative decline [91,92]. Late effects of injury or chronic, progressive musculoskeletal conditions may account for continued dominance of this diagnostic category in our study population and is also consistent with advancing age. Further investigation of the circumstances, as well as long-term outcomes of injury-related musculoskeletal conditions, would be informative, and, more importantly, could result in focused prevention strategies.

Our time-to-event modeling results were generally consistent with a previous hospitalization study of US Gulf War veterans covering the period from August 1991 to July 1999 [26]. In our study, Army personnel were more likely to be hospitalized compared with other services, even though relative proportions of active duty service members by service branch remained stable over time. There were no notable differences in the distribution of diagnostic categories for hospitalizations by service branch that might have provided additional insight.

Consistent with results from previous studies of 1991 Gulf War veterans [33,81,93] and medical surveillance reports regarding DoD-wide hospitalization rates [91,92,94,95], women were more likely to be hospitalized than men. Based on available DoD-wide data, the female-to-male crude rate ratio for hospitalizations, excluding pregnancy-related causes, was 1.6 in 2000 and 2001 and 1.5 in 2003 and 2004 for the active duty component [91,92,94,95]. In our study, adjusted rate ratios for females compared with males were similar across service branches (1.7 for Army, 1.9 for Navy, and 1.8 for Air Force), although slightly higher (2.2) for Marines. Also, independent of gender, married service members were more likely to be hospitalized. Family responsibilities or other circumstances may influence vulnerability to the experiences of war, or perhaps married individuals may be more likely to seek health care than their unmarried counterparts.

We found no increased risk of hospitalization associated with either potential exposure to nerve agents at Khamisiyah or smoke from oil well fires, again consistent with earlier hospitalization studies [26,29,33]. On the other hand, in-theater hospitalization was a predictor of long-term, postwar hospitalization across all service branches. This finding has not been previously reported. Because ascertainment of in-theater hospitalizations was not complete [28], we may have underestimated the magnitude of the association between this and subsequent long-term hospitalization. This finding may warrant further investigation.

Among those who were present in theater during ground combat, there was some variation in the risk of long-term hospitalization by service branch. Navy personnel had a slightly increased risk, while for Army and Air Force, the risk was reduced but not statistically significant. A possible explanation is that in-theater Navy personnel who go ashore tend to be elite combatants or part of construction battalions known as Seabees. Gray *et al*. reported that Seabees deployed to the Gulf War were more likely to self-report smoking, newly diagnosed digestive diseases or depression, and one or more hospitalizations since August 1990, compared with Seabees deployed elsewhere or not deployed [96].

Finally, occupation was a risk factor for long-term hospitalization only for Marines and Air Force personnel, with combat specialists having a reduced risk. We might surmise that combat specialists in the Air Force are more likely to include individuals with technologically advanced training and education, who may typify a health and safety-oriented group. Similarly, frontline Marine combatants may belong to a sub-population that selects for those with lower long-term hospitalization risk. Overall, the Marines had a lower cumulative probability of hospitalization than the other services.

The strengths of our study include 10 years of follow-up of a well-defined population of active duty service members from the 1991 Gulf War cohort with near complete ascertainment of an objective health outcome measure. On the other hand, we did not have a comparison group of non-deployed service members from the same era, nor could we ascertain hospitalizations among service members in our study cohort who separated from military service. Thus, restricting our analyses to Gulf War-deployed, active duty personnel introduced a selection bias and is a limitation of our study. Individuals with serious and debilitating injuries or illnesses following deployment would not meet fitness for duty standards, most likely leading to separation from military service. Additionally, once individuals are separated from the military, complete hospitalization data for are not readily accessible for analysis. Finally, hospitalization is only a partial measure of long-term morbidity, and other sources, such as automated ambulatory data, might provide additional insight.

## Conclusion

We provide a description of the overall long-term hospitalization experience in the subset of Gulf War veterans with military service longevity. Intra-cohort comparisons by service branch present information on the influence of Gulf War-related exposures and experiences on subsequent long-term hospitalization. Multivariable modeling suggests that in-theater hospitalization was the only significant independent predictor of long-term hospitalization risk among the selected war-related exposures or experiences examined. Although analyses were limited to Gulf War-deployed veterans who remained on active duty, our results offer some insight into the relative importance of predictors for long-term morbidity within this subset using an objective outcome measure. Additionally, this investigation adds to our understanding of retention and resiliency following large-scale combat operations.

## Competing interests

The author(s) declare that they have no competing interests.

## Authors' contributions

All authors participated in study design, data acquisition, and interpretation of the results of analyses. KB and BN performed the statistical analyses. TH, SD, KB, BN, BS, and TS helped to draft the manuscript. TH and GG made revisions to the manuscript related to intellectual content. All authors read and approved the final manuscript.

## Pre-publication history

The pre-publication history for this paper can be accessed here:



## References

[B1] Department of Defense (US) (1994). Report of the Defense Science Board Task Force on Persian Gulf War Health Effects, June 1994.

[B2] National Institutes of Health (US) Technology Assessment Workshop Panel (1994). The Persian Gulf experience and health. JAMA.

[B3] Institute of Medicine (US) (1995). Health consequences of service during the Persian Gulf war: initial findings and recommendations for immediate action.

[B4] Persian Gulf Veterans Coordinating Board (US) (1995). Unexplained illnesses among Desert Storm veterans: a search for causes, treatment, and cooperation. Arch Intern Med.

[B5] Coker WJ (1996). A review of Gulf War illness. J R Nav Med Serv.

[B6] Institute of Medicine (US) (1996). Health consequences of service during the Persian Gulf war: recommendations for research and information systems.

[B7] Presidential Advisory Committee on Gulf War Veterans' Illnesses (1996). Final Report.

[B8] The Iowa Persian Gulf Study Group (1997). Self-reported illness and health status among Gulf War veterans. a population-based study. JAMA.

[B9] (1998). Health Study of Canadian Forces Personnel Involved in the 1991 Conflict in the Persian Gulf Volume 1. Ottawa, Ontario, Canada: Goss Gilroy, Inc.

[B10] Coker WJ, Bhatt BM, Blatchley NF, Graham JT (1999). Clinical findings for the first 1000 Gulf war veterans in the Ministry of Defence's medical assessment programme. BMJ.

[B11] Ishoy T, Guldager B, Appleyard M, Suadican P, Hein HO, Gyntelberg F (1999). Health status after serving in the Gulf war area. The Danish Gulf War Study. Ugeskr Laeger.

[B12] Unwin C, Blatchley N, Coker W, Ferry S, Hotopf M, Hull L, Ismail K, Palmer I, David A, Wessely S (1999). Health of UK servicemen who served in Persian Gulf War. Lancet.

[B13] Doebbeling BN, Clarke WR, Watson D, Torner JC, Woolson RF, Voelker MD, Barrett DH, Schwartz DA (2000). Is there a Persian Gulf War syndrome? Evidence from a large population-based survey of veterans and nondeployed controls. Am J Med.

[B14] Cherry N, Creed F, Silman A, Dunn G, Baxter D, Smedley J, Taylor S, Macfarlane GJ (2001). Health and exposures of United Kingdom Gulf war veterans. Part I: The pattern and extent of ill health. Occup Environ Med.

[B15] Lee HA, Gabriel R, Bale AJ, Bolton P, Blatchley NF (2001). Clinical findings of the second 1000 UK Gulf War veterans who attended the Ministry of Defence's Medical Assessment Programme. J R Army Med Corps.

[B16] Lee HA, Gabriel R, Bolton JPG, Bale AJ, Jackson M (2002). Health status and clinical diagnoses of 3000 UK Gulf War veterans. J R Soc Med.

[B17] Smith TC, Smith B, Ryan MAK, Gray GC, Hooper TI, Heller JM, Dalanger NA, Kang HK, Gackstetter GD (2002). Ten years and 100,000 participants later: occupational and other factors influencing participation in US Gulf War health registries. J Occup Environ Med.

[B18] Hotopf M, David AS, Hull L, Nikalaou V, Unwin C, Wessely S (2003). Gulf War illness--better, worse, or just the same? a cohort study. BMJ.

[B19] Gray GC, Gackstetter GD, Kang HK, Graham JT, Scott KC (2004). After more than 10 years of Gulf War veterans medical evaluations, what have we learned?. Am J Prev Med.

[B20] Eisen SA, Kang HK, Murphy FM, Blanchard MS, Reda DJ, Henderson WG, Toomey R, Jackson LW, Alpern R, Parks BJ, Klimas N, Hall C, Pak HS, Hunter J, Karlinsky J, Battistone MJ, Lyons MJ, Gulf War Study Participating Investigators (2005). Gulf War veterans' health: medical evaluation of a U.S. cohort. Ann Int Med.

[B21] Gackstetter GD, Hooper TI, Al Qahtani MS, Smith TC, Memish ZA, Schlangen KM, Cruess DF, Barrett DH, Ryan MAK, Gray GC (2005). Assessing the potential health impact of the 1991 Gulf War on Saudi Arabian National Guard soldiers. Int J Epidemiol.

[B22] Salamon R, Verret C, Jutand MA, Begassat M, Laoudj F, Conso F, Brochard P (2006). Health consequences of the first Persian Gulf War on French troops. Int J Epidemiol.

[B23] Sim M, Kelsall H (2006). Gulf War illness: a view from Australia. Philos Trans R Soc Lond B Biol Sci.

[B24] Cowan DN, Lange JL, Heller J, Kirkpatrick J, DeBakey S (2002). A case-control study of asthma among U.S. Army Gulf War veterans and modeled exposure to oil well fire smoke. Mil Med.

[B25] Lange JL, Schwartz DA, Doebbeling BN, Heller JM, Thorne PS (2002). Exposures to the Kuwait oil fires and their association with asthma and bronchitis among Gulf War veterans. Environ Health Perspect.

[B26] Smith TC, Heller JM, Hooper TI, Gackstetter GD, Gray GC (2002). Are Gulf War veterans experiencing illness due to exposure to smoke from Kuwaiti oil well fires? Examination of Department of Defense hospitalization data. Am J Epidemiol.

[B27] Kelsall HL, Sim MR, Forbes AB, McKenzie DP, Glass DC, Ikin JF, Ittak P, Abramson MJ (2004). Respiratory health status of Australian veterans of the 1991 Gulf War and the effects of exposure to oil fire smoke and dust storms. Thorax.

[B28] Smith TC, Corbeil TE, Ryan MA, Heller JM, Gray GC (2004). In-theater hospitalizations of US and allied personnel during the 1991 Gulf War. Am J Epidemiol.

[B29] Gray GC, Smith TC, Knoke JD, Heller JM (1999). The postwar hospitalization experience of Gulf War veterans possibly exposed to chemical munitions destruction at Khamisiyah, Iraq. Am J Epidemiol.

[B30] Kang HK, Bullman TA (2001). Mortality among US veterans of the Persian Gulf War: 7-year follow-up. Am J Epidemiol.

[B31] McCauley LA, Rischitelli G, Lambert WE, Lasarev M, Sticker DL, Spencer PS (2001). Symptoms of Gulf War veterans possibly exposed to organophosphate chemical warfare agents at Khamisiyah, Iraq. Int J Occup Environ Health.

[B32] McCauley LA, Lasarev M, Sticker D, Rischitelli DG, Spencer PS (2002). Illness experience of Gulf War veterans possibly exposed to chemical warfare agents. Am J Prev Med.

[B33] Smith TC, Gray GC, Weir JC, Heller JM, Ryan MAK (2003). Gulf War veterans and Iraqi nerve agents at Khamisiyah: postwar hospitalization data revisited. Am J Epidemiol.

[B34] Bullman TA, Mahan CM, Kang HK, Page WF (2005). Mortality in US Army Gulf War veterans exposed to 1991 Khamisiyah chemical munitions destruction. Am J Public Health.

[B35] Gackstetter GD, Hooper TI, DeBakey SD, Johnson A, Nagaraj BE, Heller JM, Kang HK (2006). Fatal motor vehicle crashes among veterans of the 1991 Gulf War and exposure to munitions demolitions at Khamisiyah: a nested case-control study. Am J Ind Med.

[B36] Proctor SP, Heaton KJ, Heeren T, White RF (2006). Effects of sarin and cyclosarin exposure during the 1991 Gulf War on neurobehavioral functioning in US army veterans. NeuroToxicology.

[B37] Page WF, Mahan CM, Bullman TA, Kang HK (2005). Health effects in Army Gulf War veterans possibly exposed to chemical munitions destruction at Khamisiyah, Iraq: Part I. Morbidity associated with potential exposure. Mil Med.

[B38] Page WF, Mahan CM, Kang HK, Bullman TA (2005). Health effects in Army Gulf War veterans possibly exposed to chemical munitions destruction at Khamisiyah, Iraq: Part II. Morbidity associated with notification of potential exposure. Mil Med.

[B39] Hotopf M, David A, Hull L, Ismail K, Unwin C, Wessely S (2000). Role of vaccinations as risk factors for ill health in veterans of the Gulf War: cross sectional study. BMJ.

[B40] Shaheen S (2000). Shots in the desert and Gulf War syndrome. Evidence that multiple vaccinations during deployment are to blame is inconclusive. BMJ.

[B41] Cherry N, Creed F, Silman A, Dunn G, Baxter D, Smedley J, Taylor S, Macfarlane GJ (2001). Health and exposures of United Kingdom Gulf War veterans. Part II: The relation of health to exposure. Occup Environ Med.

[B42] Schumm WR, Reppert EJ, Jurich AP, Bollman SR, Webb FJ, Castelo CS, Stever JC, Sanders D, Bonjour GN, Crow JR, Fink CJ, Lash JF, Brown BF, Hall CA, Owens BL, Krehbiel M, Deng LY, Kaufman M (2002). Self-reported changes in subjective health and anthrax vaccination as reported by over 900 Persian Gulf War era veterans. Psychol Rep.

[B43] Tournier JN, Jouan A, Mathieu J, Drouet E (2002). Gulf War syndrome : could it be triggered by biological warfare-vaccines using pertussis as an adjuvant?. Med Hypotheses.

[B44] Macfarlane GJ, Biggs AM, Maconochie N, Hotopf M, Doyle P, Lunt M (2003). Incidence of cancer among UK Gulf war veterans: cohort study. BMJ.

[B45] Kelsall HL, Sim MR, Forbes AB, Glass DC, McKenzie DP, Ikin JF, Abramson MJ, Blizzard L, Ittak P (2004). Symptoms and medical conditions in Australian veterans of the 1991 Gulf War: relation to immunisations and other Gulf War exposures. Occup Environ Med.

[B46] Mahan CM, Kang HK, Dalager NA, Heller JM (2004). Anthrax vaccination and self-reported symptoms, functional status, and medical conditions in the National Health Survey of Gulf War Era Veterans and Their Families. Ann Epidemiol.

[B47] Kelsall H, Macdonell R, Sim M, Forbes A, McKenzie D, Glass D, Ikin J, Ittak P (2005). Neurological status of Australian veterans of the 1991 Gulf War and the effect of medical and chemical exposures. Int J Epidemiol.

[B48] Peakman M, Skowera A, Hotopf M (2006). Immunological dysfunction, vaccination and Gulf War illness. Philos Trans R Soc Lond B Biol Sci.

[B49] Bell IR, Warg-Damiani L, Baldwin CM, Walsh ME, Schwartz GE (1998). Self-reported chemical sensitivity and wartime chemical exposures in Gulf War veterans with and without decreased global health ratings. Mil Med.

[B50] Proctor SP, Heeren T, White RF, Wolfe J, Borgos MS, Davis JD, Pepper L, Clapp R, Sutker PB, Vasterling JJ, Ozonoff D (1998). Health status of Persian Gulf War veterans: self-reported symptoms, environmental exposures and the effect of stress. Int J Epidemiol.

[B51] Ishoy T, Suadicani P, Guldager B, Appleyard M, Gyntelberg F (1999). Risk factors for gastrointestinal symptoms. The Danish Gulf War Study. Dan Med Bul.

[B52] Reid S, Hotopf M, Hull L, Ismail K, Unwin C, Wessely S (2001). Multiple chemical sensitivity and chronic fatigue syndrome in British Gulf War veterans. Am J Epidemiol.

[B53] Spencer PS, McCauley LA, Lapidus JA, Lasarev M, Joos SK, Storzbach D (2001). Self-reported exposures and their association with unexplained illness in a population-based case-control study of Gulf War veterans. J Occup Environ Med.

[B54] Macfarlane GJ, Hotopf M, Maconochie N, Blatchley N, Richards A, Lunt M (2005). Long-term mortality amongst Gulf War Veterans: is there a relationship with experiences during deployment and subsequent morbidity?. Int J Epidemiol.

[B55] Sharabi Y, Danon YL, Berkenstadt H, Almog S, Mimouni-Bloch A, Zisman A, Dani S, Atsmon J (1991). Survey of symptoms following intake of pyridostigmine during the Persian Gulf War. Isr J Med Sci.

[B56] Haley RW, Kurt TL (1997). Self-reported exposure to neurotoxic chemical combinations in the Gulf War. A cross-sectional epidemiologic study. JAMA.

[B57] Kaiser KS (2000). Pyridostigmine bromide intake during the Persian Gulf War is not associated with postwar handgrip strength. Mil Med.

[B58] Nisenbaum R, Barrett DH, Reyes M, Reeves WC (2000). Deployment stressors and a chronic multisymptom illness among Gulf War veterans. J Nerv Ment Dis.

[B59] Cook MR, Graham C, Sastre A, Gerkovich MM (2002). Physiological and performance effects of pyridostigmine bromide in healthy volunteers: a dose-response study. Psychopharmacology.

[B60] Schumm WR, Reppert EJ, Jurich AP, Bollman SR, Webb FJ, Castelo CS, Stever JC, Kaufman M, Deng LY, Krehbiel M, Owens BL, Hall CA, Brown BF, Lash JF, Fink CJ, Crow JR, Bonjour GN (2002). Pyridostigmine bromide and the long-term subjective health status of a sample of over 700 male Reserve Component Gulf War era veterans. Psychol Rep.

[B61] Solomon Z, Margalit C, Waysman M, Bleich A (1991). In the shadow of the Gulf War: psychological distress, social support and coping among Israeli soldiers in a high risk area. Isr J Med Sci.

[B62] Dlugosz LJ, Hocter WJ, Kaiser KS, Knoke JD, Heller JM, Hamid NA, Reed RJ, Kendler KS, Gray GC (1999). Risk factors for mental disorder hospitalization after the Persian Gulf War: U.S. Armed Forces, June 1, 1991-September 30, 1993. J Clin Epidemiol.

[B63] Suadicani P, Ishoy T, Guldager B, Appleyard M, Gyntelberg F (1999). Determinants of long-term neuropsychological symptoms. The Danish Gulf War Study. Dan Med Bull.

[B64] Fiedler N, Lange G, Tiersky L, DeLuca J, Policastro T, Kelly-McNeil K, McWilliams R, Korn L, Natelson B (2000). Stressors, personality traits, and coping of Gulf War veterans with chronic fatigue. J Psychosom Res.

[B65] Barrett DH, Doebbeling CC, Schwartz DA, Voelker MD, Falter KH, Woolson RF, Doebbeling BN (2002). Posttraumatic stress disorder and self-reported physical health status among U.S. military personnel serving during the Gulf War period: a population-based study. Psychosomatics.

[B66] Barrett DH, Gray GC, Doebbeling BN, Clauw DJ, Reeves WC (2002). Prevalence of symptoms and symptom-based conditions among Gulf War veterans: current status of research findings. Epidemiol Rev.

[B67] Wolfe J, Proctor SP, Erickson DJ, Hu H (2002). Risk factors for multisymptom illness in US Army veterans of the Gulf War. J Occup Environ Med.

[B68] Riddle JR, Brown M, Smith T, Ritchie EC, Brix KA, Roman J (2003). Chemical warfare and the Gulf War: a review of the impact on Gulf veterans' health. Mil Med.

[B69] Hotopf M, David A, Hull L, Nikalaou V, Unwin C, Wessely S (2004). Risk factors for continued illness among Gulf War veterans: a cohort study. Psychol Med.

[B70] Ikin JF, Sim MR, Creamer MC, Forbes AB, McKenzie DP, Kelsall HL, Glass DC, McFarlane AC, Abramson MJ, Ittak P, Dwyer T, Blizzard L, Delaney KR, Horsley KWA, Harrex WK, Schwarz H (2004). War-related psychological stressors and risk of psychological disorders in Australian veterans of the 1991 Gulf War. British J Psychiatry.

[B71] Ishoy T, Knop J, Suadicani P, Guldager B, Appleyard M, Gyntelberg F (2004). Increased psychological distress among Danish gulf war veterans – without evidence for a neurotoxic background. The Danish Gulf War Study. Dan Med Bul.

[B72] McDiarmid MA, Engelhardt S, Oliver M, Gucer P, Wilson PD, Kane R, Kabat M, Kaup B, Anderson L, Hoover D, Brown L, Handwerger B, Albertini RJ, Jacobson-Kram D, Thorne CD, Squibb KS (2004). Health effects of depleted uranium on exposed Gulf War veterans: a 10-year follow-up. J Toxicol Environ, Part A.

[B73] Fiedler N, Ozakinci G, Hallman W, Wartenberg D, Brewer NT, Barrett DH, Kipen HM (2006). Military deployment to the Gulf War as a risk factor for psychiatric illness among US troops. Br J Psychiatry.

[B74] Hyams KC, Wignall FS, Roswell R (1996). War syndromes and their evaluation: from the U.S. Civil War to the Persian Gulf War. Ann Intern Med.

[B75] Wessely S (2001). Ten years on: what do we know about the Gulf War syndrome?. Clin Med.

[B76] Jones E, Hodgins-Vermaas R, McCartney H, Everitt B, Beech C, Poynter D, Palmer I, Hyams K, Wessely S (2002). Post-combat syndromes from the Boer war to the Gulf war: a cluster analysis of their nature and attrition. BMJ.

[B77] Gardner JW, Gibbons RV, Hooper TI, Cunnion SO, Kroenke K, Gackstetter G (2003). Identifying new diseases and their causes: the dilemma of illnesses in Gulf War veterans. Mil Med.

[B78] Gronseth GS (2005). Gulf War syndrome: a toxic exposure? A systematic review. Neurol Clin.

[B79] Brown M (2006). Toxicological assessments of Gulf War veterans. Philos Trans R Soc Lond B Biol Sci.

[B80] Gray GC, Kang HK (2006). Healthcare utilization and mortality among veterans of the Gulf War. Philos Trans R Soc Lond B Biol Sci.

[B81] Gray GC, Coate BD, Anderson CM, Kang HK, Berg SW, Wignall FS, Knoke JD, Barrett-Connor E (1996). The postwar hospitalization experience of U.S. veterans of the Persian Gulf War. N Engl J Med.

[B82] Knoke JD, Gray GC (1998). Hospitalizations for unexplained illnesses among U.S. veterans of the Persian Gulf War. Emerg Infect Dis.

[B83] Gray GC, Smith TC, Kang HK, Knoke JD (2000). Are Gulf War veterans suffering war-related illnesses? Federal and civilian hospitalizations examined, June 1991 to December 1994. Am J Epidemiol.

[B84] Knoke JD, Gray GC, Garland FC (1998). Testicular cancer and Persian Gulf War service. Epidemiology.

[B85] Smith TC, Gray GC, Knoke JD (2000). Is systemic lupus erythematosus, amyotrophic lateral sclerosis, or fibromyalgia associated with Persian Gulf War service? An examination of Department of Defense hospitalization data. Am J Epidemiol.

[B86] Kang HK, Bullman TA (1996). Mortality among U.S. veterans of the Persian Gulf War. N Engl J Med.

[B87] Macfarlane GJ, Thomas E, Cherry N (2000). Mortality among UK Gulf War veterans. Lancet.

[B88] Kang HK, Bullman TA, Macfarlane GJ, Gray GC (2002). Mortality among US and UK veterans of the Persian Gulf War: a review. Occup Environ Med.

[B89] (2001). DoD Occupational Conversion Index: Enlisted/Officer/Civilian.

[B90] Winkenwerder W Technical Report: Modeling and Risk Characterization of US Demolition Operations at the Khamisiyah Pit.

[B91] Army Medical Surveillance Activity (2005). Hospitalizations among active component members, US Armed Forces, 2004. Medical Surveillance Monthly Report.

[B92] Army Medical Surveillance Activity (2002). Annual Summary, US Armed Forces-2001. Hospitalizations among active duty personnel. Medical Surveillance Monthly Report.

[B93] Carney CP, Sampson TR, Voelker M, Woolson R, Thorne P, Doebbeling BN (2003). Women in the Gulf War: combat experience, exposures, and subsequent health care use. Mil Med.

[B94] Army Medical Surveillance Activity (2004). Hospitalizations among active component members, US Armed Forces, 2003. Medical Surveillance Monthly Report.

[B95] Army Medical Surveillance Activity (2001). Annual Summary, US Armed Forces-2000. Hospitalizations among active duty personnel. Medical Surveillance Monthly Report.

[B96] Gray GC, Reed RJ, Kaiser KS, Smith TC, Gastanaga VM (2002). Self-reported symptoms and medical conditions among 11,868 Gulf War-era veterans: the Seabee Health Study. Am J Epidemiol.

